# Disease in the *Pld4*^thss/thss^ Model of Murine Lupus Requires TLR9

**DOI:** 10.4049/immunohorizons.2300058

**Published:** 2023-08-09

**Authors:** Amanda L. Gavin, Tanya R. Blane, Therese C. Thinnes, Emma Gerlt, Ann Marshak-Rothstein, Deli Huang, David Nemazee

**Affiliations:** * Department of Immunology and Microbiology, The Scripps Research Institute, La Jolla, CA; † University of California, Merced, Merced, CA; ‡ University of Massachusetts Medical School, Worcester, MA; § Life Sciences Institute, Zhejiang University, Hangzhou, China

## Abstract

Phospholipase D4 (PLD4) is an endolysosomal exonuclease of ssRNA and ssDNA, rather than a phospholipase as its name suggests. Human polymorphisms in the *PLD4* gene have been linked by genome-wide association studies to systemic sclerosis, rheumatoid arthritis, and systemic lupus erythematosus. However, B6.129 *Pld4*^−/−^ mice develop features of a distinct disease, macrophage activation syndrome, which is reversed in mice mutated in TLR9. In this article, we compare a *Pld4* null mutant identified on the BALB/c background, *Pld4*^thss/thss^, which has distinct phenotypes: short stature, thin hair, and features of systemic lupus erythematosus. All phenotypes analyzed were largely normalized in *Pld4*^thss/thss^*Tlr9*^−/−^ mice. Thus, *Pld4*^thss/thss^ represents a rare model in which mouse lupus etiology is TLR9 dependent. Compared with PLD4-deficient B6 mice, *Pld4*^thss/thss^ mice had elevated levels of serum IgG, IgG anti-dsDNA autoantibodies, BAFF, and IFN-γ and elevated B cell numbers. Overall, the data suggest that PLD4 deficiency can lead to a diverse array of rheumatological abnormalities depending upon background-modifying genes, and that these diseases of PLD4 deficiency are largely driven by TLR9 recognition of ssDNA.

## Introduction

The importance of genetic background on the phenotype of mutations in mice and other organisms has been well documented (1–8). In mouse studies, null mutants are generally available on a single genetic background, such as the C57BL/6 (B6) or 129 strains, likely limiting our understanding of the potential phenotypes and their parallels to human disease. We previously carried out a classical embryonic stem cell targeting to study the function of the phospholipase D4 (*Pld4*) gene in mice. In the course of that work, we generated a floxed allele on the 129 background and backcrossed it to B6. In parallel, we generated a null mutation by introducing a ubiquitously expressed Cre transgene. We extensively characterized the knockout mice on the B6 background. The most striking and stable phenotypes included mild splenomegaly and upregulation of MHC class II (MHCII) on peritoneal macrophages as a result of elevated production of IFN-γ (9). These features led us to identify the enzymatic function of PLD4 as an endolysosomal exonuclease (9). Additional phenotypes, including liver inflammation, elevation of marginal zone (MZ) B cell numbers and blood monocytes, and reductions in numbers of B-1 B cells, NK cells, and platelets, were found (9).

In 2012, Harris et al. at Jackson Laboratories reported on its informatics Web site (http://www.informatics.jax.org/allele/reference/J:179503) the identification of a spontaneous recessive *Pld4* mutant on the BALB/c background called *thss* that was identified by thin hair and short stature, features that were not noticeable in our *Pld4*^−/−^ mice backcrossed to B6. Although the initial description indicated that both *thss* females and males are fertile and live a normal life span, they noted a less than expected 25% homozygous offspring in F2 crosses, suggesting some prenatal death.

Subsequently, Akizuki et al. (10) showed that *thss* mice had some features of lupus erythematosus, including splenomegaly, B cell expansion, anti-DNA autoantibodies, and immune complex deposits in the glomeruli. This is considered significant, because human genome-wide association studies have linked *PLD4* polymorphisms to systemic sclerosis, lupus, and rheumatoid arthritis (10–12). Moreover, systemic lupus erythematosus in particular is a notoriously heterogeneous disease with poorly understood and complex etiology and genetic control. Interestingly, among a herd of cattle arose a *Pld4* null mutation, which in homozygous form caused a phenotype somewhat reminiscent of the *Pld4*^thss/thss^ strain, including runting and skin inflammation (13), which has recently been associated with dermal macrophage activation (14). The current study was undertaken to directly compare the phenotypes of *Pld4* mutation on different mouse genetic backgrounds and to test the dependence of the disease of *Pld4*^thss/thss^ mice on TLR9.

Our prior study showed that the major phenotypes of B6.129 *Pld4*^−/−^ mice were lost when they carried a mutated *Tlr9* (9). That TLR9 mutant (*Tlr9*^CpG11/CpG11^) turned out to be a hypomorph rather than a total deficiency (15), yet completely “cured” disease. In this study, we compared embryonic stem cell–derived B6.129 *Pld4*^−/−^ mice with BALB/c *Pld4*^thss/thss^ mice. We also compared these with a CRISPR-mutagenized PLD4 null generated on the B6 background. The effect of TLR9 mutation on these phenotypes, including a nonleaky TLR9 knockout on the BALB/c background, was assessed.

## Materials and Methods

### Mice

BALB/cJ-*Pld4^thss^*/GrsrJ (BALB/c *Pld4*^thss/thss^) mice were obtained from Jackson Labs (Stock No. 012624). *Tlr9*^−/−^ mice on a BALB/c background were from Prof. Marshak-Rothstein’s UMass breeding colony. B6.*Pld4*^−/−^ and B6.*Pld4*^mut/mut^ strains were generated at The Scripps Research Institute as described later. BALB/cJ and B6 mice were obtained from Jackson Labs and maintained in our vivarium. Unless otherwise noted, in each experiment mice of both sexes were used, and experimental and control mice were age and sex matched. Animals studied were cohoused in the same animal rooms, although control and experimental strains were usually maintained in separate cages. All animal use was carried out under protocols approved by the Scripps Research Institutional Animal Care and Use Committee.

### Pld4 targeting

B6.*Pld4*^−/−^ and B6.*Pld4*^mut/mut^ strains were generated in a CRISPR/Cas9 targeting experiment using B6 zygotes. The guide RNA sequences used were 5′-GTGTTCTACACTCCAAATTC-3′ and 5′-ACTCCAAATTCTGGGTTG-3′. The targeting construct was an oligonucleotide (purchased from IDT) of the following sequence: 5′-A*A*TGACCCCCACAAGATCCTAGGCTTAAGACCCAAC*A*C-3′, in which the asterisks indicate phosphorothioate linkages, and the underlined bases are modified from wild type (WT) to alter codons encoding HSK amino acid residues in the first HKD motif to GSE and generating a new BamHI site. Mice with modified alleles were identified from ear punch DNA preparations using a PCR with the following primers: mPLD4-KI-test-F2, 5′-AGATCTATTAGGCAGTTCTGAAGTTA-3′, and mPLD4-KI-test-R1, 5′-GGTCATGTAGAACCTTCTAACTGGT-3′. After an initial denaturation at 94°C for 5 min, PCR conditions were 94°C for 30 s, 57°C for 30 s, and 72°C for 25 s for 40 cycles using the Q5 2× mastermix (NEB). The PCR products (414 bp) were then digested at bp 143 with BamH1 to identify the knock-in. A second digest with ApoI cuts adjacent to the target site in both WT and knock-in, but not in the knockout, allele D14, which has deleted 14 bp, including the HSK codons and ApoI site in the targeted exon.

### Body weight

Littermates, including male and female mice of the indicated strains, were weighed at weaning or at 5 wk of age.

### Blood analysis

Monocyte and RBC counts in whole blood containing heparin from age-matched male and female animals were determined using a IDEX Procyte DX Machine for blood subset analysis.

### Naive Ab levels

Nunc MaxiSorp ELISA plates were coated overnight with 2 μg/ml Donkey anti-mouse IgG (Jackson ImmunoResearch), before blocking with PBS/2% BSA, and addition of sera at various concentrations diluted with PBS 1% BSA. Isotype-specific Ig detection was performed using biotin-labeled anti-IgG1 (A85-1), anti-IgG2b (R12-3), and anti-IgG3 (R40-82) from BD Biosciences and anti-IgG2a (RMG2a-62) from BioLegend, before Streptavidin-HRP addition (BD Biosciences).

### Anti-DNA Ab

Nunc MaxiSorp ELISA plates were coated overnight with 5 μg/ml dsDNA from Lambda bacteriophage (New England BioLabs) using Reacti-Bind DNA Coating Solution (Thermo Scientific) according to the manufacturer’s instructions. After blocking with PBS/2% BSA, sera were diluted with PBS/1% BSA and added at various concentrations. Plates were washed with PBS/0.05% Tween 20, and bound Ab was detected using pooled biotin-labeled anti-IgGs listed earlier before Streptavidin-HRP addition (BD Biosciences). Anti-RNA Abs were determined as described earlier; only Baker’s Yeast RNA (Sigma) was used to coat the plate.

### Flow cytometry analysis

All flow cytometry methods were essentially carried out as previously described, using comparable gating strategies and reagents (9). Intracellular staining was used to determine PLD4 expression (Alexa 647–labeled P4-16.3 Ab [in-house]) and TLR9 expression (PE-labeled J15A7 clone from BD Biosciences). In brief, after surface Ag stains, cells were fixed with 3% formaldehyde in PBS for 10 min, washed with PBS, then permeabilized in PBS supplemented with 1% FCS, 2 mM EDTA, and 0.05% Saponin (PB). All intracellular staining (PLD4 and TLR9) was done in the presence of saponin for 1 h at room temperature before washing with PB. After the final wash, cells were resuspended in PBS supplemented with 1% FCS, 2 mM EDTA.

### Serum cytokine analysis

Analysis of serum levels of BAFF was carried out with Mouse BAFF Quantikine ELISA kit (R&D Systems) sera diluted 1:10 according to the manufacturer’s instructions. Analysis of levels of IFN-γ in sera was determined using FlowCytomix beads (eBioscience) with sera diluted 1:2.

### IgG and C3 deposition in kidney

Kidneys from 6-mo-old female animals were sliced in half with a scalpel blade, submerged in OCT (Sakura), and frozen over liquid nitrogen. Six-micrometer sections were cut on a cryostat, and sections were fixed in ice-cold acetone for 10 min. Sections were blocked with PBS containing 1% bovine serum for 30 min, before overnight staining for either mouse IgG with a 1/500 dilution Alexa 488 goat anti-mouse IgG Ab (Jackson Immunoresearch) or complement using a 1/500 dilution of goat anti-mouse C3c Ab (Nordic-MUbio catalog number GAM/C3c). After washing in PBS, the complement stain was revealed using Alexa 488 Bovine anti-goat IgG (1/500; Jackson Immunoresearch). Slides were mounted with Prolong Diamond anti-fade (ThermoFisher) and sealed with nail polish. Images were taken using a Keyence microscope, and the scale shown is 100 μm.

### Statistical methods

Statistical analysis used an unpaired two-tailed *t* test for pairwise comparisons (Prism 9; GraphPad). Significant *p* values are displayed in the figures.

## Results

### Short stature of thss mice is reversed in the absence of TLR9

**FIGURE 1.**
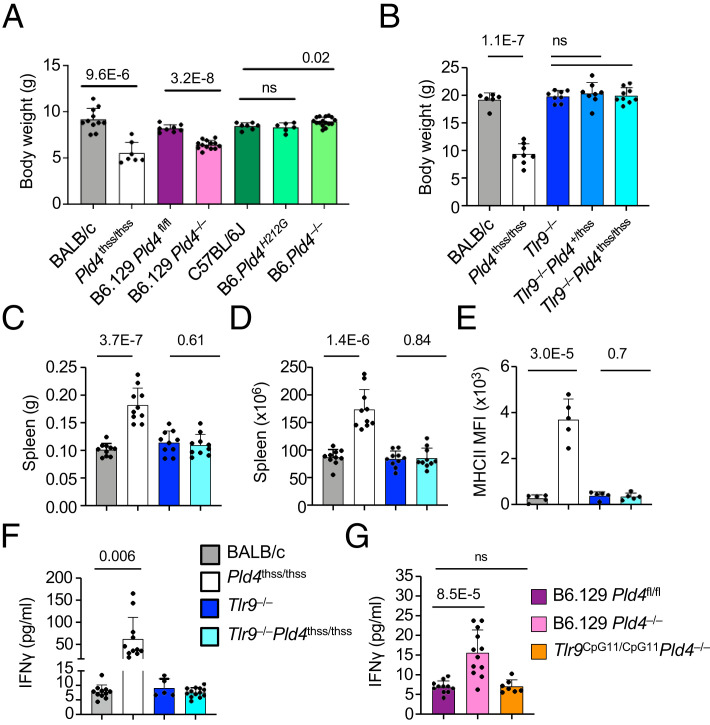
Analysis of the effect of PLD4 deficiency and genetic background on mouse body weight and TLR9 dependence of splenomegaly and MHCII levels in *Pld4*^thss/thss^ mice. Mice of the indicated genotypes were assessed for body weight at (**A**) 17 d old and (**B**) 5 wk of age. In (A), *Pld4*^thss/thss^ were on the BALB/c background. In (B), all animals were on the BALB/c background. (**C** and **D**) Analysis of the effect of TLR9 codeficiency on *Pld4*^thss/thss^ splenomegaly by weight (C) and cell number (D). (**E**) Effect of TLR9 codeficiency on MHCII levels of *Pld4*^thss/thss^ peritoneal macrophages. Gating was first on forward scatter height versus width for doublet removal; then MHCII levels were assessed on CD11b^+^F480^+^ gated cells. (**F** and **G**) Analysis of serum IFN-γ levels in PLD4-deficient mice on the BALB/c (F) or B6 (G) backgrounds and the effect of TLR9 deficiency. Each data point indicates the value obtained from an individual mouse. In (C)–(G), mice of the indicated genotypes were compared at the age of 2 mo. Significant differences as determined by two-tailed unpaired *t* test are indicated.

A key phenotype of *Pld4*^thss/thss^ mice is their strikingly short stature, which is apparent at an early age (http://www.informatics.jax.org/reference/J:171492) (10). To determine whether this phenotype is driven by TLR9, we bred *Pld4*^thss/thss^ mice to a TLR9 knockout on the BALB/c background and assessed body weight. As expected, *Pld4*^thss/thss^ mice weighed significantly less than WT BALB/c mice: ∼60% of normal at day 17 (*p* < 10^−5^) and 49% at day 35 (*p* < 10^−6^) (Fig. 1A, 1B). We observed no strong sex bias for body-weight reduction (Supplemental Fig. 1), nor any other parameter described later (Supplemental Table I). Strikingly, the runting phenotype was completely normalized by codeficiency in TLR9 (Fig. 1B). Interestingly, *Pld4*^−/−^ mice generated on a B6 background by CRISPR mutation (B6.*Pld4*^−/−^) showed no such weight reduction compared with control B6 mice (Fig. 1A, compare columns 5 versus 7). Similarly, a HKD site-directed mutant on the B6 background that expressed nonfunctional PLD4 was no lighter than control (B6.*Pld4*^mut/mut^; Fig. 1A, column 6). However, the B6.129 *Pld4*^−/−^ strain had an intermediate phenotype, weighing 78% of its B6.129 *Pld4*^fl/fl^ congenic control (Fig. 1A, columns 3 and 4). We note that the *Pld4* gene lies on the tip of chromosome 12 near the *Igh* locus, and the B6.129 *Pld4*^−/−^ strain retains the 129-derived Igh^a^ allotype, which lies ∼500 kb away. We conclude that the severe runting phenotype of *Pld4*^thss/thss^ mice requires both the BALB/c background and functional TLR9.

### Reversal of splenomegaly and MHCII elevation on peritoneal macrophages by Tlr9 codeficiency

Analysis of *Pld4*^thss/thss^*Tlr9*^−/−^ mice revealed that the splenomegaly characteristic of *Pld4*^thss/thss^ mice was completely normalized in the absence of TLR9, as measured by spleen weight and counts of nucleated cells (Fig. 1C, 1D). These results are similar to the phenotype normalization seen in the B6.129 *Tlr9*^CpG11/CpG11^*Pld4*^−/−^ strain compared with TLR9-sufficient B6.129 *Pld4*^−/−^ (9).

B6.129 *Pld4*^−/−^ mice have very high expression of MHCII on peritoneal macrophages, which was shown to be caused by a PLD4 deficiency in CD11c^+^ cells that in turn drove IFN-γ production (9). *Pld4*^thss/thss^ mice also exhibit elevated MHCII levels on peritoneal macrophages that was normalized in *Pld4*^thss/thss^*Tlr9*^−/−^ mice (Fig. 1E). Measurement of serum IFN-γ levels revealed a 7-fold elevation in *Pld4*^thss/thss^ mice compared with BALB/c controls and a return to WT levels in *Pld4*^thss/thss^*Tlr9*^−/−^ mice (Fig. 1F). The 7-fold increase in serum IFN-γ levels was significantly higher than that seen on the B6.129 background, where the increase was ∼2-fold (62.2 versus 15.6 pg/ml; *p* = 0.003, two-tailed *t* test; Fig. 1G). We conclude that PLD4 deficiency in *Pld4*^thss/thss^ mice resulted in increased IFN-γ secretion with attendant MHCII upregulation on peritoneal macrophages, and that these phenotypes required TLR9.

### Alterations in the B cell compartment, anti-DNA Abs, and correlation with BAFF

**FIGURE 2.**
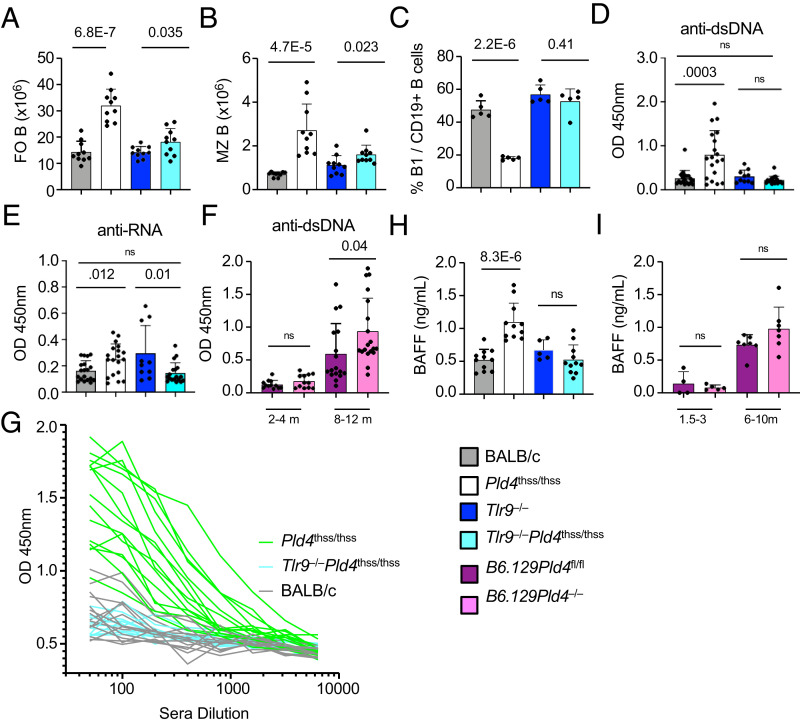
Analysis of naive B cell subsets, anti–nucleic acid autoantibodies, and the effect of TLR9 codeficiency in *Pld4*^thss/thss^ mice. (**A**) Splenic follicular B cell (FO B) numbers per spleen. Singlet live cells, identified by forward light scatter and negativity of GV510 uptake, were gated as Ter119^−^CD93^−^B220^+^CD19^+^CD23^+^. (**B**) Splenic MZ B cell numbers per spleen. Singlet live cells, identified by forward light scatter and negativity of GV510 uptake, were gated as Ter119^−^CD93^−^B220^+^CD19^+^CD23^lo^CD21^hi^. (**C**) Peritoneal B-1 B cell (B1) frequencies, calculated as percent of CD19^+^ cells. Singlet cells were gated as B220^+^CD19^+^ (B2) or B220^lo^CD19^+^ (B1). Each data point indicates the value obtained from an individual mouse. (**D** and **E**) Anti-dsDNA titers (D) and anti-RNA titers (E), as determined by ELISA at 1/200 serum dilution. Each data point indicates the value obtained from an individual mouse at 2–4 mo of age. (**F**) Comparison of anti-dsDNA titers in B6.129*Pld4*^fl/fl^ compared with B6.129*Pld4*^−/−^ mice at 2–4 mo versus 8–12 mo of age, demonstrating the comparatively lower levels in this strain in young adults. (**G**) ELISA data shown at a range of serum dilutions for a number of individual mice at 2–4 mo of age of the indicated genotypes, BALB/c, *Pld4*^thss/thss^, and *Pld4*^thss/thss^*Tlr9*^−/−^ mice. Each line represents the values obtained from one mouse. (**H** and **I**) Analysis of serum BAFF levels in mice of the indicated genotypes. Bars show mean ± SD. Significant differences, as determined by two-tailed unpaired *t* test, are indicated.

In agreement with Akizuki et al. (10), *Pld4*^thss/thss^ mice had increased numbers of follicular “B2” B cells and MZ B cells, respectively, 225% and 391%, compared with BALB/c controls. We found that these abnormalities were also dependent on TLR9, because they were largely normalized in *Pld4*^thss/thss^*Tlr9*^−/−^ mice (Fig. 2A, 2B), although there was a residual elevation in B2 and MZ numbers, 28% and 42%, compared with *Tlr9*^−/−^ controls. Interestingly, follicular B cell hyperplasia was not consistently observed in B6.129 *Pld4*^−/−^ mice as compared with B6.129 *Pld4*^+/−^ controls (figure S1h in Ref. 9). Significantly higher MZ B cell numbers were observed in B6.129 *Pld4*^−/−^ mice, a phenotype that was previously shown to be TLR9 dependent (9). Conversely, in the peritoneum, B-1 B cell proportions were reduced, a phenotype that was reversed in *Pld4*^thss/thss^*Tlr9*^−/−^ mice (Fig. 2C, compare columns 2 and 4). Overall, some B cell phenotypes caused by PLD4 deficiency are shared and others differ depending upon strain background.

We verified that young (2- to 4-mo-old) *Pld4*^thss/thss^ mice produce significantly elevated anti-dsDNA Abs compared with BALB/c controls (Fig. 2D), whereas we were unable to detect appreciable anti-RNA titers (Fig. 2E). Data in Fig. 2D show values obtained at serum dilution of 1/200, but similar results were obtained at a range of concentrations (Fig. 2G). In contrast, sera from 2- to 4-mo-old B6.129 *Pld4*^−/−^ mice lacked appreciable anti-DNA compared with B6.129 *Pld4*^fl/fl^ controls (Fig. 2F), although at 8–12 mo of age there was a trend to higher levels (right bars). Importantly, the anti-dsDNA autoantibody production in *Pld4*^thss/thss^ was fully TLR9 dependent because titers were normalized in sera of *Pld4*^thss/thss^*Tlr9*^−/−^ mice, which were comparably low with *Tlr9*^−/−^ or BALB/c controls (Fig. 2D).

Features of B cell hyperplasia and autoantibodies can be indicative of elevated levels of the B cell survival cytokine BAFF (TNFSF13b) (16), which have been detected in *Pld4*^thss/thss^ mice (10). We confirmed that *Pld4*^thss/thss^ mice have about double the BAFF levels of BALB/c controls (Fig. 2H) and further show that this BAFF elevation is reversed in *Pld4*^thss/thss^*Tlr9*^−/−^ mice and is absent in young adult B6.129 *Pld4*^−/−^ mice (Fig. 2H, 2I). These data support a model in which the selective B cell hyperplasia and autoantibody formation in *Pld4*^thss/thss^ mice is promoted by higher levels of BAFF.

### Anti-DNA Abs

**FIGURE 3.**
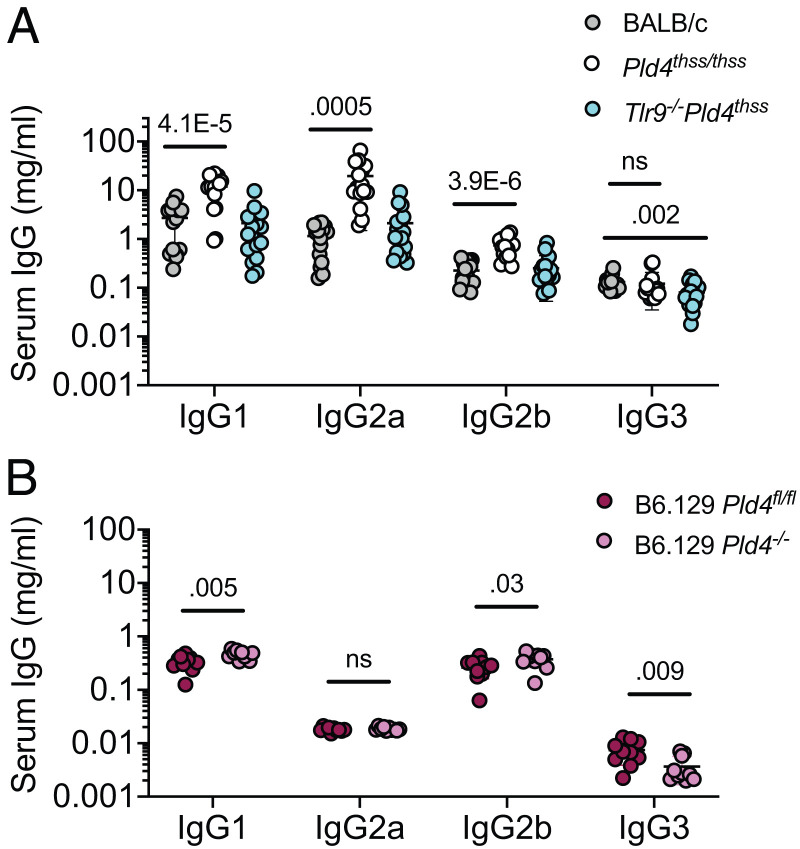
Analysis of normal serum Ig levels in indicated mouse strains. (**A**) Comparison of Ig levels in BALB/c, *Pld4*^thss/thss^, and *Pld4*^thss/thss^*Tlr9*^−/−^ sera. (**B**) Comparison of Ig levels in B6.129*Pld4*^fl/fl^ versus B6.129*Pld4*^−/−^ mice at 2–4 mo. Each data point indicates the value obtained from an individual mouse. Significant differences, as determined by two-tailed unpaired *t* test, are indicated.

We also measured total serum Ig levels in the strains in question. *Pld4*^thss/thss^ mice had IgG1, IgG2a, and IgG2b levels ∼4-, 17-, and 3-fold higher than BALB/c controls, respectively (Fig. 3A). Importantly, serum levels were normalized in *Pld4*^thss/thss^*Tlr9*^−/−^ mice (Fig. 3A, turquoise dots). Although B6.129 *Pld4*^−/−^ mice showed marginally significant elevations compared with B6.129 *Pld4*^fl/fl^ controls in IgG1 and IgG2b, mean levels were <50% higher (Fig. 3B). The particularly elevated IgG2a levels in *Pld4*^thss/thss^ mice may be partly explained by their much higher IFN-γ levels (Fig. 1F). Thus, PLD4 deficiency on the BALB/c background is associated with significantly elevated B cell function, whereas this is muted in the B6 background.

### Alterations in the NK compartment

**FIGURE 4.**
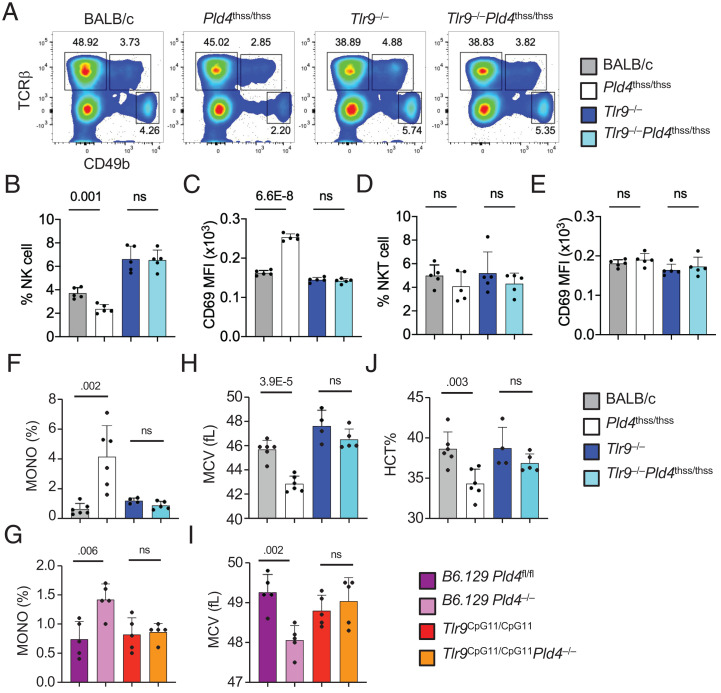
TLR9-dependent alterations in NK cells, monocytes, erythrocytes, and platelets of PLD4-deficient mice. (**A**–**D**) Reduced NK cell proportions with elevated NK cell CD69 expression in *Pld4*^thss/thss^ mice and normalization in *Pld4*^thss/thss^*Tlr9*^−/−^ mice. The indicated mouse strains, BALB/c, *Pld4*^thss/thss^, *Tlr9*^−/−^, and *Pld4*^thss/thss^*Tlr9*^−/−^ mice, were analyzed for the frequency of NK and NKT cells and the relative expression levels of CD69 on these cell subsets. (A) Flow cytometry gating strategy used, involving costaining with Abs against TCRβ and CD49b. (B) NK cell frequency. (C) CD69 levels on NK cells. (D) NKT cell frequency. (**E**) CD69 levels on NKT cells. (**F**–**J**) Analysis of the hematological abnormalities of monocytosis and anemia in the indicated mouse strains: (F, H, and J) BALB/c, *Pld4*^thss/thss^, *Tlr9*^−/−^, and *Pld4*^thss/thss^*Tlr9*^−/−^ mice on the BALB/c background. (G and I) *Pld4*^fl/fl^, *Pld4*^−/−^, *Tlr9*^CpG11/CpG11^, and *Tlr9*^CpG11/CpG11^*Pld4*^−/−^ mice on the B6 background. Whole blood samples from 2- to 4-mo-old mice were analyzed using an IDEX Procyte DX Machine. (F and G) Counts of monocytes from the indicated strains as a percentage of leukocytes. (H and I) Mean RBC volume. (J) Hematocrit. Each data point indicates the value obtained from an individual mouse. All bar graphs show mean ± SD. Significant differences, as determined by two-tailed unpaired *t* test, are indicated.

We previously reported that NK cell proportions were reduced by ∼50% in B6.129 *Pld4*^−/−^ mice compared with B6.129 *Pld4*^fl/fl^ controls, and that this decrease was independent of IFN-γ but dependent upon TLR9 (9). We therefore assessed NK cell numbers in *Pld4*^thss/thss^ mice along with *Pld4*^thss/thss^*Tlr9*^−/−^, *Tlr9*^−/−^, and BALB/c control mice. NK cells are defined as TCRβ^−^CD49^++^, whereas NKT cells are TCRβ^+^CD49^+^ (Fig. 4A). We found a statistically significant reduction in NK cell proportions in *Pld4*^thss/thss^ mice compared with BALB/c controls (*p* = 0.001, *n* = 5/group; Fig. 4B). Importantly, NK cells were restored in *Pld4*^thss/thss^*Tlr9*^−/−^ mice (Fig. 4B, turquoise bar). The remaining NK cells in *Pld4*^thss/thss^ mice showed higher levels of CD69 suggesting activation (Fig. 4C), an increase that was also normalized in the absence of TLR9. NKT cell numbers analyzed in the same samples were not significantly different (Fig. 4D), nor did the NKT cells express more CD69 (Fig. 4E).

### Changes in blood monocytes

Elevation in blood monocytes is another feature of B6.129 *Pld4*^−/−^ mice that appears to be replicated in *Pld4*^thss/thss^ mice. Quantitated as the frequency among blood leukocytes, *Pld4*^thss/thss^ mice had an increase of >4-fold compared with BALB/c controls (Fig. 4F), which represented an even larger increase than in the B6.129 *Pld4*^−/−^ strain (Fig. 4G). Monocyte levels were normalized in *Pld4*^thss/thss^*Tlr9*^−/−^ mice (Fig. 4F) [as was the case in B6.129 *Pld4*^−/−^*Tlr9*^CpG11/CpG11^ mice, in which the *Tlr9* gene carries a missense mutation that retains minimal function detectable in some assays (15)]. Other TLR9-driven alterations in blood of *Pld4*^thss/thss^ mice included reduced hematocrit and a slightly smaller mean RBC volume (Fig. 4H, 4J). These features were also shared with B6.129 *Pld4*^−/−^ mice (Fig. 4I) (9).

### Alterations in spleen neutrophils and monocyte/macrophage marker expression

**FIGURE 5.**
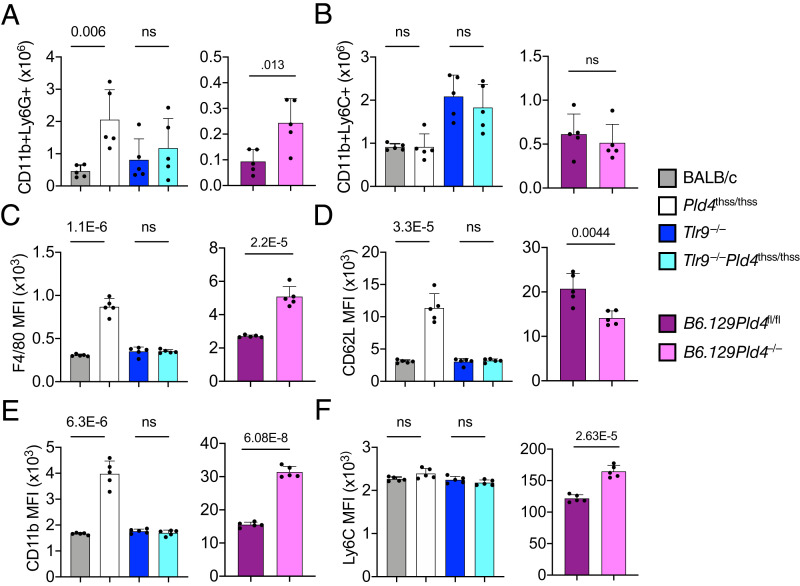
Analysis of changes in numbers of CD11b^+^Ly6G^+^ splenic neutrophils and surface phenotype of CD11b^+^Ly6C^+^Ly6G^−^ splenic monocyte/macrophages. (**A**) Elevated CD11b^+^Ly6G^+^ splenic neutrophil cell numbers in PLD4-deficient mice. (**B**) Lack of change in CD11b^+^Ly6C^+^Ly6G^−^ splenocyte cell numbers in PLD4-deficient mice. (**C**–**F**) Analysis of changes in marker expression level in CD11b^+^Ly6C^+^Ly6G^−^ splenocytes of PLD4-deficient mice. Neutrophils were gated as forward scatter singlet GV510^−^Ter119^−^CD11b^+^Ly6G^hi^; monocyte/macrophage was gated as forward scatter singlet GV510^−^Ter119^−^CD11b^+^Ly6G^−^Ly6C^hi^. Data show mean fluorescence intensity (MFI) of the markers indicated on the vertical axis. Each data point represents the value obtained in independent mice of the indicated strains. Plots show mean with SD indicated by error bars. Significant differences, as determined by two-tailed unpaired *t* test, are indicated.

BALB/c *Pld4*^thss/thss^ and B6.129 *Pld4*^−/−^ mice both had increased numbers of splenic neutrophils compared with PLD4-sufficient controls (Fig. 5A). This increase was again dependent upon TLR9 (Fig. 5A). In contrast, the numbers of CD11b^+^Ly6C^+^Ly6G^−^ splenic myeloid cells were similar in PLD4-deficient and -sufficient mice (Fig. 5B). However, CD11b^+^Ly6C^+^Ly6G^−^ splenic myeloid cells from PLD4-deficient mice exhibited alterations in the intensity of expression of particular cell surface markers, including F4/80 and CD11b (Fig. 5C, 5D). There were two strain-specific differences between BALB/c *Pld4*^thss/thss^ and B6.129 *Pld4*^−/−^ CD11b^+^Ly6C^+^Ly6G^−^ splenocytes compared with PLD4-sufficient controls, with CD62L highly elevated in BALB/c *Pld4*^thss/thss^ cells, whereas Ly6C was higher in B6.129 *Pld4*^−/−^. Although the function of Ly6C is obscure, CD62L promotes homing to secondary lymphoid tissues.

### Analysis of TLR9 and PLD4 levels in B cells and dendritic cells of BALB/c and B6 mice

**FIGURE 6.**
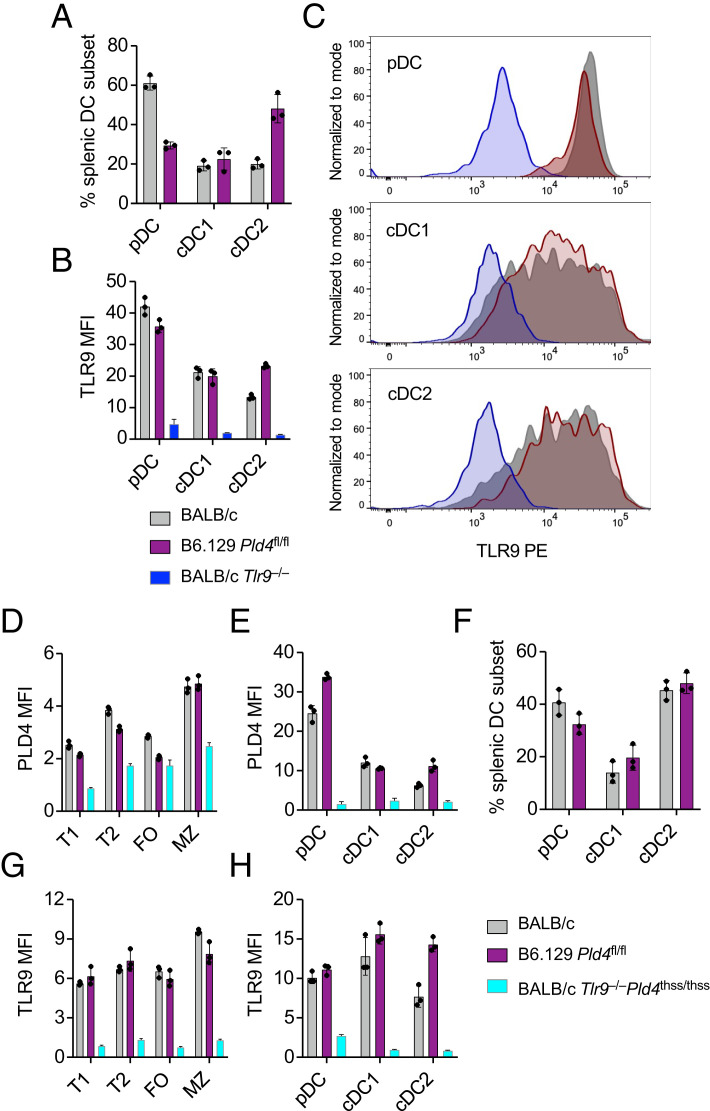
Comparative expression of TLR9 and PLD4 in subsets of B cells and DCs from B6 and BALB/c mice. (**A**–**C**) Flow cytometry analysis of DCs. (A) Proportions of splenic DC subtypes obtained. (B) Comparative TLR9 expression (MFI) in DCs of B6 and BALB/c mice, using BALB/c *Tlr9*^−/−^ DCs as negative control. (C) Histogram shows sensitivity of the assay. (**D**–**H**) Analysis of TLR9 and PLD4 expression of DCs and B cells from B6 and BALB/c mice. PLD4 expression in (D) B cell subsets and (E) DC subsets. (F) DC subset proportions found in this experiment. TLR9 expression in (G) B cell subsets and (H) DC subsets, using similarly gated BALB/c *Pld4*^thss/thss^*Tlr9*^−/−^ splenocytes as negative control. Flow cytometry gating strategy for this experiment is given in Supplemental Fig. 2.

We were able to measure TLR9 and PLD4 levels in B cell and dendritic cell (DC) subsets of splenocytes using flow cytometry (Fig. 6). Although the overall pattern of TLR9 expression was similar, B6 cDC2 cells appeared to have somewhat higher levels of TLR9 than BALB/c cDC2 cells (Fig. 6B, 6C, 6H). The expression patterns of PLD4 broadly agreed with mRNA databases, with DCs expressing much higher levels than B cells (Fig. 6D, 6E). Control staining of cells from BALB/c *Tlr9*^−/−^*Pld4*^thss/thss^ mice confirmed the specificity of the Abs and also confirmed that *Pld4*^thss/thss^ cells are indeed deficient in PLD4 protein.

### Kidney pathology

**FIGURE 7.**
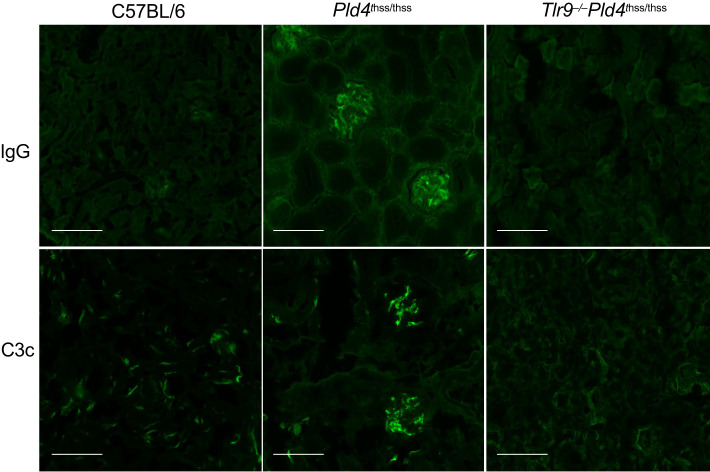
Immunofluorescence analysis of kidney sections in *Pld4*^thss/thss^ compared with *Pld4*^thss/thss^*Tlr9*^−/−^ mice. Frozen sections from kidneys of 6-mo-old females of the indicated mouse strains were stained for IgG and C3 deposition. Scale bars, 100 μm.

*Pld4*^thss/thss^ mice have been reported to show some aspects of kidney pathology associated with lupus, although their long-term survival is not affected (10). We confirmed the observation that *Pld4*^thss/thss^ mice have increased IgG and activated complement C3 deposition in the kidneys. Importantly, these anomalies were absent in BALB/c *Pld4*^thss/thss^*Tlr9*^−/−^ mice (Fig. 7).

## Discussion

Because anti-dsDNA autoantibodies are diagnostic of lupus erythematosus, it has long been hypothesized that DNA sensing in general, and TLR9 in particular, might play a role in disease (17). But in mice, numerous studies have shown that TLR7 signaling is central to the lupus phenotype (18, 19), whereas knockout of TLR9 alone has only subtle effects on autoantibody specificity or exacerbated disease (20–28). This is somewhat puzzling because TLR7 and TLR9 are thought to signal similarly through MyD88 and TASL and are often, but not always, coexpressed in the same cells (29, 30). In contrast, dysregulated but functional TLR9 mutants may develop hemophagocytic lymphohistiocytosis–like symptoms (31). That TLR9 deficiency reverses all of the phenotypes of *Pld4*^thss/thss^ mice indicates that this strain is a rare murine lupus model that is mainly TLR9 driven. This is a striking and unexpected outcome. For example, mice deficient in the extracellular DNase, DNase1L3, develop late-life lupus and anti-dsDNA that is dependent upon MyD88 (32). In that case, the syndrome is the result of deficiency in a nuclease specific for DNA, so one might have expected a mainly TLR9-driven disease. However, when *Dnase1L3*^−/−^ mice were rendered TLR9 deficient, only a modest amelioration in disease was observed, whereas TLR7/9 double-deficient mice were protected (24). In this study, the PLD4 nuclease cleaves both RNA and DNA substrates, and small RNA and DNA oligonucleotides accumulate in PLD4-deficient mice (9, 15), yet loss of a sensor for DNA alone was able to normalize the phenotype of PLD4 deficiency. Although we cannot exclude a contribution of TLR7, which would require the generation of *Pld4*^thss/thss^*Tlr7*^−/−^ mice on the BALB/c background, *Pld4*^thss/thss^*Tlr9*^−/−^ mice appear to be completely normal phenotypically, apart, of course, from the inability to signal through TLR9. One explanation for the different TLR dependence between typical mouse lupus models and *Pld4*^thss/thss^ mice may be that the former is driven by RNA, presumably derived from endogenous retrovirus to stimulate TLR7 in endolysosomes, whereas the latter is presumably mainly driven mainly by excess ssDNA in endolysosomes. In humans, lysosomal DNA overload might also be the driver for a significant subset of patients.

*Pld4*^thss/thss^ mice have hair and size phenotypes not seen in B6.*Pld4*^−^*^/^*^−^ mice, suggesting that background affects phenotype. The simplest hypothesis for the difference is that, on the BALB/c background, PLD3 expression fails to compensate for lack of PLD4 in certain key cell types. Alternatively, other nucleases may fail to compensate. However, we have so far not uncovered any evidence for this among mouse strain-specific RNA sequencing databases. Our working hypotheses are that osteoclasts, a macrophage-like cell responsible for bone resorption, contribute to *Pld4*^thss/thss^ mouse skeletal problems, whereas DCs are aberrantly activated in *Pld4*^thss/thss^ skin, because these cell types both normally express PLD4 and can be activated by TLR9 (9, 33, 34). In any case, because *Pld4*^thss/thss^*Tlr9*^−/−^ mice lack these features, it appears that these phenotypes are also TLR9 driven. These data in turn support the hypothesis that PLD4 function in TLR9-expressing cells is critical for the observed phenotypes, although other possibilities are not excluded.

A major distinction between PLD4-deficient mice on the BALB/c background compared with B6 is the development of lupus-like features and B cell hyperreactivity. Follicular and MZ, but not B-1, B cell survival are dependent on levels of BAFF. Excess BAFF also can promote plasma cell survival and lead to elevated Ab (and autoantibody) levels (16). For DNA-reactive B cells in particular, alterations in BAFF levels can have remarkable effects on tolerance escape (35). The elevated levels of BAFF in PLD4-deficient mice on the BALB/c compared with B6 background likely explain these differences and point to a difference in TLR9-driven BAFF production in these two backgrounds. Elevated IFN-γ is a feature of lupus that has also been implicated in driving BAFF production by myeloid cells (36), possibly suggesting that the extraordinarily high IFN-γ levels in *Pld4*^thss/thss^ mice might promote the BAFF elevation in this strain.

In addition to possible differences in TLR9 expression levels in key cell types, TLR9 itself differs between BALB/c and B6. Mills et al. (37) showed that the BALB/c allelic version of TLR9 signals less strongly in B cells, leading to an autoantibody phenotype when in genetic combination with the CD45^E613R^ “wedge” mutation. Stronger signaling by the B6 version of TLR9 instead promoted B cell death at the transitional stage, suggesting a dose-dependent contribution to B cell tolerance. That TLR9 might contribute to B cell death or tolerance has been proposed in other contexts (31, 38–41). However, TLR9 ligands can also suppress receptor editing (42). Although the B cell compartment appears to be differentially affected in *Pld4*^thss/thss^ compared with B6.129*Pld4*^−^*^/^*^−^ mice, it is not known whether these differences are secondary to myeloid cell activation or are instead B cell autonomous.

The anemia, blood monocytosis, and splenic neutrophilia of *Pld4*^thss/thss^ mice were also TLR9 dependent and shared with PLD4-deficient mice on the B6 background. These phenomena are possibly related, because altered erythrocyte features are likely secondary to myeloid cell activation and hemophagocytosis (43).

The lupus-like disease of *Pld4*^thss/thss^ mice is somewhat atypical. *Pld4*^thss/thss^ mice manifest early-age reduction in stature (runting), yet survive long term despite kidney IgG and C3 deposition, and there is only a subtle sex bias (http://www.informatics.jax.org/allele/reference/J:179503) (10). Nonetheless, genome-wide association studies show *PLD4* is strikingly associated with human systemic lupus erythematosus and dsDNA Abs diagnostic of lupus (10). This suggests that PLD4 insufficiency along with attendant TLR9 triggering may promote lupus disease in some patients, suggesting that they might benefit from treatments that blockade or inhibit TLR9 or its downstream pathways.

In summary, PLD4 deficiency can lead to a diverse array of abnormalities, a subset of which is distinct in the BALB/c compared with B6 backgrounds. Although PLD4 is both an RNase and a DNAse, the phenotypes driven by deficiency seem to work entirely through TLR9 on both genetic backgrounds. The explanations for this are still not entirely clear. One possibility is simply that there are many more redundant RNases than there are DNases, or that RNA is inherently less stable. The nature of TLR7/8 signaling may also play a role because for these sensors, extensive processing, down to single nucleosides, is required for activation (44, 45). Hence absence of PLD4 may hinder, rather than enhance, signaling to some substrates, as we recently observed in myeloid cells lacking both PLD3 and PLD4 (15). However, the strain-specific differences in pathology in PLD4-deficient mice are quite striking, and their understanding will require further investigations. In any case, our findings imply that human PLD4 polymorphism and deficiency may have diverse phenotypic presentations ranging from hemophagocytic lymphohistiocytosis to lupus, and that all may be treatable by blockade of TLR9.

## Supplementary Material

Supplemental 1 (PDF)

Supplemental 2 (XLS)
