# Supplementary material for: Disease in the Pld4thss/thss Model of Murine Lupus Requires TLR9
Source: Immunohorizons. 2023 Aug 9;7(8):577–86. doi: 10.4049/immunohorizons.2300058 (PMC10441812; doi:10.4049/immunohorizons.2300058)
Supplement: Supplemental 1 (PDF) [file IH_2300058_Supplemental_1.pdf]

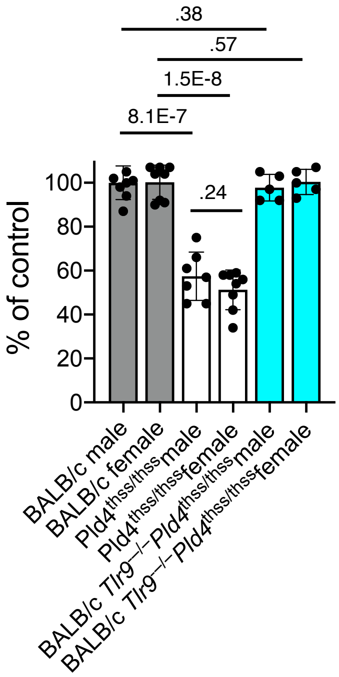

Supplementary Figure 1. Sex bias analysis of body weight in *Pld4*$^{thss/thss}$ mice. Data are calculated using BALB/c value of indicated sex as 100% control. Data from main Fig 1A,B were separated based on sex of mice. Bars indicate p values of indicated dataset pairs. See also Supplementary Table 1 for more details.

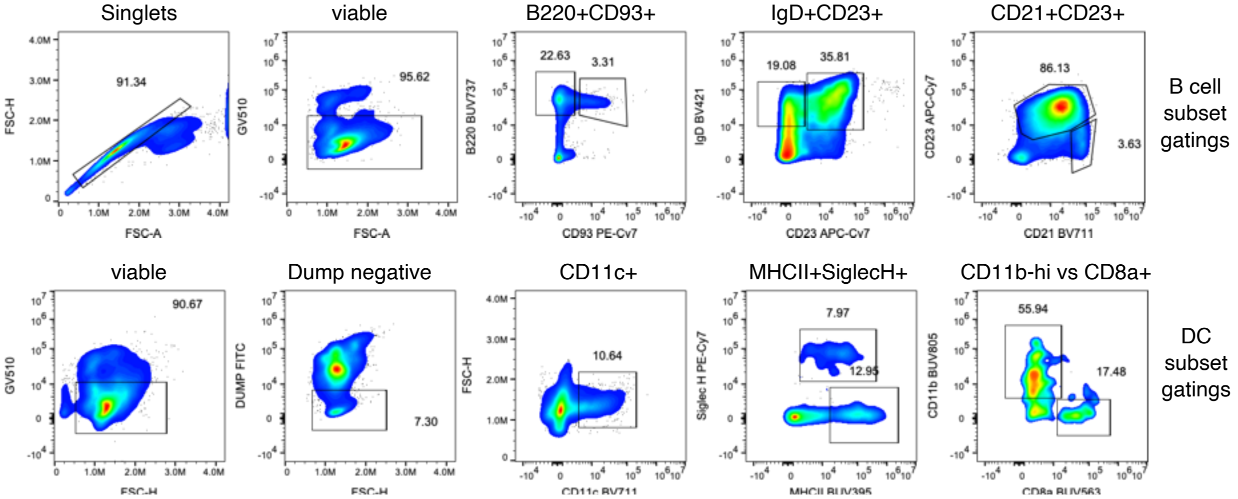

Supplementary Figure 2. Flow cytometry gating of B cell and DC subsets indicated in Figure 6.
Upper panels: show the sequence of gates employed to define the different B cell subsets. Transitional B cell subsets are gated as B220+CD93+: T1, $IgM^{hi}IgD^{-}CD21^{-}CD23^{-}$; T2, $IgM^{+}IgD^{+}CD23^{+}$; T3, $IgM^{lo}IgD^{+}$. Follicular B cells are defined as $B220^{+}CD93^{-}CD21^{+}CD23^{+}$. MZ B are $B220^{+}CD93^{-}CD21^{hi}CD23^{lo}$.
Lower panels: DCs are defined as CD11c+ and lacking in lineage markers TCRb, F4/80, CD19. pDC are additionally Siiglec H+. CD8a and CD11b distinguish cDC1 from cDC2.
